# Symmetry, presumptions, and the judges design

**DOI:** 10.1371/journal.pone.0340446

**Published:** 2026-01-29

**Authors:** Murat C. Mungan

**Affiliations:** Texas A&M University School of Law, Fort Worth, Texas, United States of America; Uniwersytet Jagiellonski w Krakowie, POLAND

## Abstract

An instrumental variables approach called ‘the judges design’ used frequently in social sciences relies on an assumption called ‘average monotonicity’. This assumption pertains to how different judges’ (or other classifiers’) decision making processes relate to each other. Violations of it are hard to detect, which raises the importance of it being supported by a plausible theory. Decisions of judges who solve Bayesian decision problems violate average monotonicity as long as the signals they process are symmetric and they do not possess strong presumptions. This result is extended to cases where judge presumptions are symmetrically distributed and may include strong presumptions. The analysis reveals factors that can be considered while discussing the plausibility of an assumption made to identify causal effects whose violations are difficult to detect and has important policy implications.

“In other cases, however where instrumental variables are used [monotonicity is] not so plausible. Specifically in what are now called judge lenience designs”

Guido Imbens,

2021 Nobel Prize Lecture

## 1 Introduction

Instrumental variables (IVs) are used frequently in empirical studies in social sciences to sidestep endogeneity problems. In part of the literature, researchers have turned to judge propensities, e.g., judges’ tendencies to return harsh verdicts, as an instrument to study the relationship between judge decisions and other outcomes of interest. This approach, often called ‘the judges design’, has been used to study causal relationships within a very wide range of topics, including, the relationship between pretrial detention and crime [1]; the effects of disability insurance and employment [2]; patents and innovation [3] and [4]; evictions and hospital visits [5]; and psychotherapy and suicide attempts [6], just to name some examples. (See [7] for a lengthier review of articles employing this approach.) Therefore, assessing and understanding the validity and the limits of this approach carries great importance, since results obtained in this literature may have great policy impact.

In this article, I provide a theoretical analysis of a component that is central to this approach, namely the relationship between judge decisions. Most of the literature using the judges design makes assumptions regarding the relationship between judge decisions to justify the causal interpretation of their results. These assumptions are violated unless a condition called *average monotonicity,* explained further below, holds (see [8] for a recent review of this literature). Within a standard Bayesian decision model, I show that when noisy signals received by judges to make decisions are *symmetric* and judges do not have strong *presumptions*, average monotonicity is violated. This makes it problematic to interpret results obtained from judges designs when these conditions hold. A brief review of the judges design approach (see, also, [9] for a lengthier review) is useful to provide background prior to explaining the three key concepts (symmetry, presumptions, and average monotonicity) in this article.

In the judges design approach, the researcher is interested in the relationship between how a subject is classified (*treated* or not; e.g., detained or not; insured or not; evicted or not) and an outcome of interest (e.g., future crime; employment; health condition). However, the classification is made by a decision maker (a *judge*), who may make the classification, in part, based on factors that are also related to the outcome of interest, which can make inference difficult. For instance, one cannot infer the impact of detention on future crime by directly comparing the recidivism rates of people who have been detained and released, respectively. This is because judges may base their detention decisions on factors correlated with defendants’ likelihood of committing crimes in the future. Studying the relationship between the treatment and the outcome directly through conventional methods is therefore problematic. Instead, one can compare the average outcomes of subjects who were randomly assigned to a judge with a high treatment rate (a *harsh* judge) and a judge with a low treatment rate (a *lenient* judge), and attribute the difference in outcome to the treatment status of the subjects.

Under conditions listed below, a local average treatment effect (LATE) can be obtained by using this approach. The LATE is the effect of treatment on the outcomes of *marginal* subjects, i.e., only those subjects whose treatment status would be different when assigned to the harsh judge and when assigned to the lenient judge. In many cases, the researcher is able to exploit variation across more than two judges. In these cases, the judges’ design can be used to obtain a positive weighted average of the LATEs associated with different pairs of judges. Four conditions are typically assumed to ensure that the IV estimates obtained from a judges’ design can be interpreted as a positive weighted average of LATEs: (i) random judge assignment, (ii) nontrivial instrument, (iii) exclusion, and (iv) monotonicity.

The first three assumptions are rather straightforward (see, e.g., [8]), and I assume they hold to focus on the fourth. (The first requires that subjects be randomly assigned to judges. Nontrivial instruments only requires that there be some variation in the treatment rate, i.e., the propensity, of judges. The third requires that judges’ only be able to affect the outcomes of subjects through their decisions regarding the subjects. This rules out other, direct, effects which judges may have on subjects, e.g., by calling them, and giving them encouragement or otherwise helping them.)

*Monotonicity* requires that a subject who is treated would have also been treated if she were assigned to a harsher judge. This assumption ensures that the difference in the number of subjects treated by any two judges equals the number of marginal subjects. This is crucial, since the researcher can only observe the number of subjects treated by each judge, and cannot observe whether a subject is marginal with respect to the two judges in question. Thus, when monotonicity holds, the ratio between the difference in the average of outcomes and the average propensities of two judges with different propensities (often called the *Wald ratio*) equals the LATE.

This assumption is often called *strict* monotonicity, as it requires there to be a clear and consistent ranking between judges in terms of the harshness of their decisions. Because this assumption is likely to be violated in a variety of settings, researchers have sought to identify weaker conditions that can replace it. Recently, ([7] p. 255) has shown that under *average monotonicity* “the instrumental variables estimator converges to a proper weighted average of individual treatment effects". Average monotonicity requires only “that each person’s potential treatment status as a function of judge assignment must be positively correlated with judge propensity" ([7] p. 269). Thus, it is a weaker requirement than strict monotonicity and its violation implies violations of strict monotonicity.

An important problem is that violations of monotonicity, whether strict or average, cannot be directly observed in most cases. This is because the researcher is ordinarily only able to observe the treatment status of the subject under the judge to whom she is randomly assigned, and cannot observe her treatment status in counterfactuals where she is assigned to other judges. Thus, at best, one can design tests that can detect violations of implications of average monotonicity.

In fact, [10] recently proposed such a test, and documented violations of average monotonicity by employing it. Even their test, however, is able to detect average monotonicity violations by focusing on judges’ misclassification rates (e.g., doctors’ failure to diagnose patients). This makes it difficult to detect average monotonicity violations generally due to two important reasons. First, in many cases misclassification rates are unobservable to the researcher, which makes it impossible to employ the test. Second, the test focuses on whether the potential treatment status of *groups of subjects* as a function of judge assignment (e.g., patients who were incorrectly not diagnosed), as a whole, is positively correlated with judge propensity, whereas average monotonicity requires this correlation for each subject individually.

These problems make average monotonicity violations difficult to detect, and thus raise the importance of developing a deeper theoretical understanding of the conditions under which average monotonicity is likely to be violated. This is especially true, since undetected violations of average monotonicity can lead to very misleading interpretations, including claims that treatment causes the opposite of the actual effect (i.e., the IV estimand and the causal effects having opposite signs; see [11, 12], and [8]). These ‘sign reversals’ and misleading interpretations can emerge even when the proportion of cases violating monotonicity is low, unless the heterogeneity in treatment effects, which are unobservable, are within certain bounds. (This is noted in the recent literature, e.g., [13], discussed below. Thus, without placing arbitrary bounds on the heterogeneity of treatment effects, one cannot rule out sign reversals. Example 1 in Sect 2, below, formalizes this point further.)

Here, I consider one of the most extensively studied decision problems, namely a two-state and two-action Bayesian decision problem, which has been used in the judges’ design literature to formulate the hypotheses to be tested (see, e.g., [14] and [10]). In this model, each judge (pronoun *he*) makes a judgment based on some imperfect information (a *signal*) that he receives regarding the subject. I show that average monotonicity is violated as long as (i) signals are *symmetric*, and (ii) judges do not hold strong *presumptions*.

Signal symmetry is a form of signal neutrality assumed frequently in the literature. In intuitive terms, signals are symmetric when the manner in which information about the presence and absence of a condition is generated in an identical manner. In more formal terms, it refers to the strength of signals supporting either state of the world being identically distributed under those respective states. An important property of symmetric signals is that they do not, on their own, tilt judges’ decisions in one direction or the other. Many, if not most, signal structures considered in the literature satisfy this requirement. The most well-known example is one where the signal is distributed normally with equal variance and differing means conditional on type. Another is the symmetric binary signal where there are only two signal realizations, e.g., up or down. When the binary signal is symmetric, ‘up’ is observed as frequently in one state of the world as ‘down’ is observed in the other state of the world. (See, e.g., [15] and the sources cited therein for further discussion of these signal structures.)

Presumptions, on the other hand, refer to which judgment judges would make, if they had not received a signal. This is consistent with the usage of the word in describing perhaps the most well-know presumption in the legal setting, namely the presumption of innocence in criminal law: lack of evidence implies an acquittal. On the other hand, a judge holds no presumptions, if he is indifferent between two judgments absent further evidence. When judges do have presumptions, they may be of differing strength, which is measured by how strong the evidence favoring the opposite state must be for the judge’s presumption to be overcome. Whether judges have presumptions, and if so how strong their presumptions are, of course, depend on the context.

As noted, when the signals received by judges are symmetric, and judges do not hold strong presumptions, average monotonicity is violated. For an intuitive explanation of this result, consider a simple example wherein judges make pretrial detention decisions. They may either detain or release defendants, and their objective is to detain only those who would commit some pretrial misconduct (e.g., not showing up at trial, or committing another crime before their trial) if released. These defendants are called type *P* defendants to abbreviate descriptions; and the defendants who would not commit pretrial misconduct are called type *N* defendants. Thus, judges may commit two types of errors: (i) Detain a type *N* person (called a *type-1 error*); and (ii) release a type *P* person (called a *type-2 error*).

Judges adopt decision criteria, which determine the probabilities with which they make these errors, conditional on the defendant’s type. These probabilities can be described as Pr(release|P) and Pr(detention|N) for clarity. When judges receive information from type *P* and type *N* defendants which are indicative of their types in the same manner, the signals received by judges are said to be symmetric. Thus, with symmetric signals, judges can adopt decision criteria which allow them to make type-1 and type-2 errors in an interchangeable way: if (*a*,*b*) denotes the type-1 and type-2 error probabilities associated with one decision criterion, signal symmetry implies that the judge can also implement another decision criterion which leads to the error probability pair (*b*,*a*). Moreover, when judges have no presumptions, they are willing to trade-off type-1 and type-2 error probabilities at an even rate, because absent the signal they are indifferent between making either decision. Thus, they adopt decision criteria which lead them to equate the conditional type-1 and type-2 error probabilities associated with their decisions.

Judges may nevertheless differ in the frequency with which they make erroneous judgments, because they may interpret and observe the information they receive from defendants differently. Thus, when the majority of the defendants are type *P*, it follows that judges who make errors less frequently end up detaining more defendants; i.e., their propensity to detain is greater. As a result, type *N* defendants are less likely to be detained when they are assigned to judges with greater propensities. The opposite holds, i.e., type *P* defendants are less likely to be detained when they are assigned to judges with greater propensities, when the majority of defendants are type N. In both cases, there is a violation of average monotonicity, which requires, as noted above, “that each person’s potential treatment status as a function of judge assignment must be positively correlated with judge propensity” ([7] p. 269). (Example 1, below, provides a more formal illustration of this insight.)

These results naturally extend to cases where judges have weak presumptions. However, when judges differ greatly in their presumptions, average monotonicity may hold, if these differences in presumptions lead to variations in judge decisions that are greater than the variations caused by differences in the way judges perceive and interpret information. Thus, whether judges hold strong presumptions is an important factor in determining whether average monotonicity holds. (It is important to note that large differences in presumptions do not necessarily translate into large differences in judge decisions. This may happen, for instance, when the marginal rate of transformation between the two errors allowed by the signal is roughly constant for a large range of error pairs. This issue is explored in further detail in Sect 3.2 and Appendix B in Supporting information. Thus, average monotonicity may be violated even with very large variations in presumptions.) Since average monotonicity (or even stronger) conditions are assumed in research employing the judges design, it becomes important to discuss whether there are plausible reasons for judges to possess strong presumptions in the institutional settings studied. This is especially so, given the difficulty of detecting violations of average monotonicity discussed, above. One way to supplement these discussions is through bounding exercises regarding how strong presumptions must be to alleviate concerns regarding average monotonicity violations. Sect 3, below, discusses how these exercises can be conducted after formalizing the relevant comparison between variations in judge decisions caused by how they process information versus their presumptions.

These observations complement recent attempts to extrapolate the potential impacts of monotonicity violations from cases in which multiple judges’ decisions on the same case are observable, but they do not individually dictate the outcome of a case. Specifically, [13] measures monotonicity violations committed by individual judges voting in panels. Because multiple judges vote on the same case, their behavior in each case, as well as their propensities are observable, which makes monotonicity violations observable. In the settings studied, the author finds that about 4% to 11% cases may be violating average monotonicity. Making some restrictive assumptions, the author argues that hypothetical judges’ designs based on these judges’ individual decisions would not generate large biases due to monotonicity violations. (In particular, the author assumes judges’ individual voting behavior mimicks their panel voting behavior –an assumption that is contested in prior literature and discussed by the author–, and he considers hypothetical heterogenous treatment effects to conduct specific calculations.)

Quite interestingly, the data analyzed in [13] come from criminal settings where judges are likely to possess a strong presumption of innocence, which may make average monotonicity violations less suspect, as explained here. Thus, extending the approach in [13] to other settings where judge presumptions are not as predictable may be particularly fruitful; and when this is not feasible, it may be useful to obtain evidence regarding judge presumptions through other means (e.g., surveys or incentivized experiments).

In addition to [7], in which average monotonicity is proposed, this article is also closely related to [10], who have shown that the decisions of the radiologists (to diagnose a patient with pneumonia based on X-Rays) they studied clearly violate average monotonicity. In fact, Chan et al. demonstrate that radiologists who tend to commit false diagnoses more frequently also fail to diagnose more frequently. They note that this type of relationship can only exist if there is decision-quality-heterogeneity across radiologists. In related work, [16] considers a model in which judges not only make decisions based on noisy signals, but also can make investments to reduce the amount of noise. In this set up, [16] shows that the behavioral pattern in [10] can emerge even when judges differ only with respect to how much they value avoiding errors, rather than their raw talent. Thus, [10] identify decision–quality-heterogeneity as a factor that *can*, but need not, lead to violations of average monotonicity, while [16] shows that these heterogeneities can be caused by preferences alone. Neither article, however, identifies a set of general conditions under which judges will behave in a manner that violates average monotonicity. Here, I propose signal symmetry and lack of strong presumptions as sufficient conditions.

In the next section, I describe a simple Bayesian model and use it to formalize the results described above. In Sect 3, I describe how one can specify bounds on the distributions of judge presumptions which may give rise to average monotonicity violations. I provide brief concluding remarks in Sect 4. Appendices provided in the accompanying supplementary file contain proofs and technical derivations.

## 2 Model and analysis

In this section I model judge decisions as solutions to simple Bayesian problems wherein judges choose a decision criterion to trade-off two possible types of decision errors. Judges receive signals which are imperfectly informative of subjects’ types, and choose a cut-off likelihood ratio (corresponding to a critical signal realization) as their decision criterion (as explained in Sect 2.1). Judges can differ in the way they observe and interpret information, modeled through different signals received by judges. In Sects 2.1 through 2.4, I consider the information structure and behavior of a single judge, and introduce the notation to consider multiple judges and the relationships between their decisions in Sect 2.5. In Sect 2.6, I extend the analysis to cases where judges may possess different presumptions; some strong and some weak.

### 2.1 The information structure

A judge (pronoun *he*) is tasked with returning *judgments* v∈{p,n} for subjects who are of one of two *types* T∈{P,N} where the letters stand for positive and negative. Types are unobservable to the judge. The proportion of type *P* people among the entire population of subjects is μ∈(0,1). The judge’s belief about this proportion (his prior) $\hat{\mu }\in (0,1)$ may or may not be accurate. Judgments and types are labeled such that matching letters indicate a *correct* match, in the sense that the judge prefers these matches over their alternatives. Table 1 summarizes the phrases that can be used to refer to each judgment-type combination and clarifies how the two errors (type-1 and -2) are defined.

**Table 1:** True/false positives and negatives.

		Subject Type (*T*)	
		P	N
Judge Decision (*v*)	*p*	True positive	False positive (Type-1 Error)
	*n*	False negative (Type-2 Error)	True negative

The judge reviews some evidence, a random variable *X*, which may be multi-dimensional, and whose distribution depends on the subject’s type. In particular, each realization *x* of the evidence *X* is associated with a likelihood ratio *l*, where the realization probability conditional on *P* is in the numerator, i.e., $\frac{\mathrm{Pr}(x|P)}{\mathrm{Pr}(x|N)}$. Thus, the likelihood ratio provides information to the judge when updating his beliefs regarding the subject’s type, and is itself a random variable, denoted *L*. I call *L* the ‘signal’ and *X* the evidence to distinguish the concepts. The state-contingent cumulative distributions of *L* are F(l|T), with the associated probability density functions f(l|T), assumed to be continuous and differentiable for convenience, with $\frac{f(l|P)}{f(l|N)}=l$. (The analysis can be extended to the case where *L* does not have continuous support. However, this exercise does not reveal additional insights and comes at the expense of additional notation and expositional inconvenicence.)

Focusing on *L* or *X* as the signal is equivalent in this context, because the judge’s optimal decision criterion is a threshold likelihood ratio, i.e., the judge returns a *p*-judgment only if the evidence realization has a sufficiently high likelihood ratio (see (3) and the accompanying explanation below). Using *L* instead of *X* in the analysis simplifies the exposition since it is unidimensional, and its support is an interval on the real line $I\subseteq {\mathbb{R}}_{0}^{+}$.

### 2.2 Judge preferences and presumptions

The judge is assumed to be an expected utility maximizer who forms beliefs through Bayes’ rule. The judge’s utility depends on the subject’s type and the judgment combinations depicted in Table 2, and are given as follows:

**Table 2:** Judge utility.

		Subject Type (*T*)	
		P	N
Judge Decision (*v*)	*p*	*b*_*p*_	−*c*_*p*_
	*n*	−*b*_*n*_	*c*_*n*_

where $b_{i},c_{i}>0\;$for i∈{p,n}. Thus, the judge’s expected utility from rendering a judgment of *p* is:

$$u_{p}=\mathrm{Pr}(P|l)b_{p}-\mathrm{Pr}(N|l)c_{p}$$ (1)

$$=\frac{\hat{\mu }f(l|P)}{\hat{\mu }f(l|P)+(1-\hat{\mu })f(l|N)}b_{p}-\frac{\left(1-\hat{\mu })f(l|N\right)}{\hat{\mu }f(l|P)+(1-\hat{\mu })f(l|N)}c_{p}$$

where the subjective probabilities Pr(T|l) are obtained via Bayes’ rule using the judge’s prior $\hat{\mu }$. Similarly, the judge’s expected utility from making an *n* decision is

$$u_{n}=-\mathrm{Pr}(P|l)b_{n}+\mathrm{Pr}(N|l)c_{n}$$ (2)

$$=-\frac{\hat{\mu }f(l|P)}{\hat{\mu }f(l|P)+(1-\hat{\mu })f(l|N)}b_{n}+\frac{\left(1-\hat{\mu })f(l|N\right)}{\hat{\mu }f(l|P)+(1-\hat{\mu })f(l|N)}c_{n}$$

and therefore he weakly prefers a judgment of *p* if, and only if, $u_{n}\leq u_{p}$, which is equivalent to

$$l^{*}\equiv c\frac{1-\hat{\mu }}{\hat{\mu }}\leq \frac{f(l|P)}{f(l|N)}=l$$ (3)

where

$$c\equiv \frac{c_{p}+c_{n}}{b_{p}+b_{n}}$$ (4)

denotes the cost of making a mistake when the subject is type *N* relative to the cost of making a mistake when the subject is type *P*. Thus, *c* captures the judge’s relative preferences.

The condition expressed in (3) reveals that the judge, indeed, uses a threshold rule, as noted in the discussion of the information structure in Sect 2.1. This threshold rule, in turn, is closely related to the judge’s *presumptions*, which I define as what judgment he would return, absent any signal. Formally, without a signal, the judge’s expected utilities from judgments *p* and *n* are, respectively,

$$u_{p}=\hat{\mu }b_{p}-(1-\hat{\mu })c_{p}\text{; and }u_{n}=-\hat{\mu }b_{n}+(1-\hat{\mu })c_{n}$$ (5)

and therefore the judge weakly prefers the judgment *p* if $u_{p}\geq u_{n}$ which is equivalent to

$$l^{*}=c\frac{1-\hat{\mu }}{\hat{\mu }}\leq 1$$ (6)

Thus, a judge holds a presumption of *P* when l^*^<1, and a presumption of *N* when l^*^ > 1. On the other hand, the judge holds no presumption when $l^{*}=1$. This last possibility can arise when the judge has preferences favoring one decision and priors favoring the other, and in a manner such that the two considerations balance each other out (i.e., $c=\frac{\hat{\mu }}{1-\hat{\mu }}\text{⧸}=1$).

When the judge has a presumption in either direction, it is also possible to conceptualize the strength of his presumption in an intuitive way. Specifically, the weakest signal realization favoring the opposite direction which would be needed to rebut the judge’s presumption is a good measure of the *strength of his presumption*. For instance, when the judge has a presumption of *N*, he would need to confront a signal realization which is at least l^*^ > 1 times more likely to be produced when the person is type *P* rather than type *N* (i.e., $\frac{f(l|P)}{f(l|N)}>l^{*}$) to render a judgment against his presumptions. Similarly, when the judge has a presumption of *P*, the weakest signal realization favoring *N* needed to rebut the judge’s presumption is $1/l^{*}$ times more likely to be produced when the person is type *N* rather than type *P*. Thus, a good measure of the strength of the judge’s presumption ought to measure $l^{*}$ and $1/l^{*}$ equally, and be increasing in $l^{*}$ for all $l^{*}>1$. A simple function that has this property is $\left|\ln(l^{*})\right|$, and thus any function that has this property will be a monotonic transformation of $\left|\ln(l^{*})\right|$. Thus, I adopt $\left|\ln(l^{*})\right|$ as a measure of the strength of the judge’s presumption, which I use to ease the proof of Corollary 1, below; and to provide a compact discussion of presumption distributions when conducting a bounding exercise in Sect 3.

**Definition1. (Presumptions).** The judge has a presumption of T∈{P,N} if, absent a signal, he strictly prefers judgement *T* over the alternative. The strength of the judge’s presumption is measured by $\rho (l^{*})=|\ln(l^{*})|$. Thus, the judge has no presumptions, i.e., $\rho (l^{*})=0$, when $l^{*}=1$.

Inspecting (3) reveals that the judge’s decision criterion is entirely driven by his presumptions. Presumptions, in turn, are a product of the judge’s preferences and his prior. It is worth stressing the relationship between these three concepts. Preferences are represented by *c*, which essentially captures how much the judge values avoiding one type of error (namely a false positive) compared to making an error of the other kind (namely a false negative). Therefore, when *c* > 1, the judge *favors* avoiding one false positive to avoiding one false negative. However, judges are often unable to trade-off one false positive for one false negative. For instance, when the judge has no information besides his prior, all he can do is make a positive classification, and expect to be wrong with a probability of $1\;-\;\hat{\mu }$; or make a negative classification, and expect to be wrong with a probability of $\hat{\mu }$. Thus, presumptions describe what a judge would do, if he had to trade-off the two types of errors, without information besides his priors. Presumptions can therefore act as tools that dictate, or determine, the default position of a decision maker, absent further evidence. This is consistent with one of the most well-known presumptions in decision making, namely ‘the presumption of innocence’, according to which a defendant must be acquitted absent evidence of guilt.

To summarize, both preferences and presumptions relate to marginal rates of substitution. The difference between preferences and presumptions is that the former describes what a judge would do if he could trade-off the two types of errors with a one-to-one conversion rate; whereas presumptions describe what he would do if he had to trade them off with a $1-\hat{\mu }$ -to-$\hat{\mu }$ conversion rate.

It may also be useful to consider how the concept of ‘bias’ may be defined or used in this framework. The word bias is sometimes used to describe some kind of prejudice in favor or against certain objects or individuals. Using this conception, judges with c⧸=1 may be said to possess bias, since this represents cases where the judge has an inherent preference over one outcome or the other. However, if the word is used to describe unequal standards being used in the decision making process, one may say that judges with $l^{*}\text{⧸}=1$ are biased, because they require stronger signals for one judgment compared to the other. Finally, the word bias may be used to describe judges who possess inaccurate priors, i.e., $\hat{\mu }\text{⧸}=\mu$. This brief discussion highlights the importance of exercising care in using the word bias to describe decision makers; in this article, I refrain from using it to avoid ambiguities.

The judge’s presumptions combined with the manner through which he observes and interprets evidence (i.e., f(l|P) and f(l|N)) together determine the judgment probabilities, which are explained, next.

### 2.3 Judgment probabilities

The probability that the judge erroneously returns a *p* judgment, when the subject is type *N* is

$$\mathrm{Pr}(p|N)=1-F(l^{*}|N)$$ (7)

which is defined as a type-1 error in Table 1.

On the other hand, the probability that the judge erroneously returns an *n* judgment, when the subject is type *P* is

$$\mathrm{Pr}(n|P)=F(l^{*}|P)$$ (8)

which is defined as a type-2 error in Table 1.

Using these observations, the probabilities of various judgments can be summarized by the following ‘confusion matrix’ (Table 3).

**Table 3:** Confusion matrix.

		Subject Type (*T*)	
		P	N
Judge Decis. (*v*)	*p*	True positives $\mu (1-F(l^{*}|P))$	False positives $\left(1-\mu )(1-F(l^{*}|N)\right)$
	*n*	False negatives $\mu F(l^{*}|P)$	True negatives $\left(1-\mu )F(l^{*}|N\right)$

Thus, the judge’s unconditional *p*-judgment probability, or propensity to return a *p* judgment can be defined as

$$\Psi (l^{*})=\mu (1-F(l^{*}|P))+(1-\mu )(1-F(l^{*}|N))$$ (9)

Next, I explain how the manner in which the judge receives and interprets evidence affects the type-1 and type-2 errors that he commits.

### 2.4 Signal symmetry and implications

The evidence reviewed by the judge, as well as his ability to interpret evidence, can both affect the accuracy of his decisions. All factors relevant to this evidence generation and interpretation process are summarized by the signal *L*, defined through the distributions f(.|P) and f(.|N). In this context, the ‘strength of the signal realization’ favoring *P* is captured by the likelihood ratio $l=\frac{f(l|P)}{f(l|N)}$. On the other hand, the strength of the signal realization favoring *N* is captured by the inverse likelihood ratio $\frac{1}{l}=\frac{f(l|N)}{f(l|P)}$. To make this relationship more explicit, let

$$\tilde{L}\equiv \frac{1}{L}$$ (10)

denote the inverse likelihood ratio. The conditional distributions of this inverse likelihood ratio can then be defined analogously to the distribution of the likelihood ratio *L*, as follows:

$$\tilde{F}(\tilde{l}|T)=\mathrm{Pr}\left(\tilde{L}\leq \tilde{l}|T\right);\text{and}$$ (11)

$$\tilde{f}(\tilde{l}|T)=\frac{d\tilde{F}(\tilde{l}|T)}{d\tilde{l}}$$

where $\tilde{l}$ is used to indicate realizations of the inverse likelihood ratio $\tilde{L}$ to distinguish them from realizations of *L* for which the symbol *l* is used.

Quite importantly, *F* describes the manner in which evidence indicative of the state *P* is distributed, whereas $\tilde{F}$ describes the manner in which evidence indicative of *N* is distributed. Symmetry is obtained when realizations of the signal that favor each of the two states, and to different degrees, are equally likely to be observed in their respective states. More simply stated, symmetry holds when the manner in which a state produces information about itself does not depend on the identity of that state. This is formalized as follows.

**Definition 2. (Symmetry).** The signal is symmetric if

$$F(y|P)=\tilde{F}(y|N)\text{ for all }y\text{.}$$ (12)

The intuition behind this definition is that state *P* produces evidence of various strength in its favor just as frequently as state *N* produces evidence of the same strength in its favor. Many signals studied in the literature in fact satisfy symmetry. For instance, the binormal distribution, where a decision maker receives signals drawn from normal distributions with equal variance but differing means under the two different states of the world is a clear example. Another example is the symmetric binary signal wherein there are only two evidence realizations, X={−1,1}, and the likelihood of one realization being observed conditional on state *P* is equal to the other realization being observed in state *N*, i.e., Pr(1|P)=Pr(−1|N). More generally, an obvious sufficient condition for symmetry is for the evidence *X* to be distributed symmetrically under the two states of the world. For instance, when X=[0,1], the signal is symmetric if γ(x|P)=γ(1−x|N) for all x∈[0,1] where γ(.|T) is the probability density of *X* conditional on state *T*. Similarly, the signal is symmetric when it has the familiar form $X=\theta_{T}+\varepsilon$ where ε is an error term symmetrically distributed around 0, and $\theta_{T}$ is the state-dependent mean of the signal.

An important property of signal symmetry is that it removes the evidence generating process as a source of unequal error production by a decision maker, i.e., the signal does not orient the decision maker in a particular direction. This orientation property has recently been proposed in [15], and can have important implications in a variety of contexts. In the current analysis, it implies that a judge who receives a symmetric signal can commit unequal errors, only if he holds a presumption. This implication is formalized, next.

**Proposition 1. (Equal errors).** If the signal is symmetric and the judge holds no presumptions, he commits equal type-1 and type-2 errors, i.e.,

$$F(l^{*}|P)=1-F(l^{*}|N)$$ (13)

*Proof*: When judges have no presumptions, it follows that $l^{*}=1$. Thus,


$$F(l^{*}|P)=F(1|P)=\tilde{F}(1|N)$$

where the second equality follows from the symmetry of signals. Next, note that


$$\tilde{F}(1|N)=\mathrm{Pr}\left(\frac{f(l|N)}{f(l|P)}\leq 1|N\right)=\mathrm{Pr}\left(\frac{f(l|P)}{f(l|N)}\geq 1|N\right)=1-F(1|N)$$

$$=1-F(l^{*}|N)$$

where the last equality follows because the judge holds no presumptions. Thus, $F(l^{*}|P)=1-F(l^{*}|N)$. □

The intuition behind proposition 1 is closely related to the implications of judges having no presumptions and perceiving symmetric signals. When judges lack presumptions they require the same signal strength in favor of the two states to make a decision favoring that state (i.e., $l^{*}={\tilde{l}}^{*}=\frac{1}{l^{*}}=1$). Signal symmetry ensures that the likelihood of mistakes given these equal cut-offs are also equal under the two states (i.e., F(1|P)=1−F(1|N)). Thus, a judge who has no presumptions, and receives symmetric signals, views the two labels (positive and negative), as completely interchangeable, and has no reason to discriminate between them. As a result, he adopts a decision criterion that causes him to make equal errors.

Next, I consider the implications of signal symmetry with respect to the relationship between different judges’ decision making processes.

### 2.5 Multiple judges and average monotonicity violations

I consider *J* > 1 judges who may differ from each other with respect to their preferences and priors as well as the manner through which they observe and interpret evidence. Variations in the latter are captured by allowing judge-specific distribution functions *f*_*j*_(*l*|*T*) where j∈{1,2,...,J} denotes a specific judge. Variations in judge preferences and priors are similarly captured by letting $l_{j}^{*}$ denote the judge-specific decision criterion, which is directly affected by judges’ preferences *c*_*j*_ and priors ${\hat{\mu }}_{j}$, obtained by allowing *c* (defined in (??)) and $\hat{\mu }$ to vary across judges. If multiple judges share the same characteristics, i.e., have the same presumptions l^*^ and receive the same signal, I collectively consider them to be a single judge to abbreviate the analysis.

The analysis focuses only on two types of subjects, T∈{P,N}, i.e., it abstracts from covariates. The presence of covariates can lead to additional sources of average monotonicity violations (e.g., judges having multi-dimensional preferences and using covariate dependent thresholds), and therefore abstracting from them allows me to focus on violations stemming from signal symmetry and lack of presumptions. This abstraction is harmless for purposes of demonstrating violations of average monotonicity (see definition 3, below), since this property is required to hold for all subjects. In fact, the analysis could be interpreted as focusing on two types of subjects who share the same covariates. These subjects, whose size is normalized to 1, are randomly assigned to judges, where *w*_*j*_ is the share of subjects assigned to judge *j*.

The probability with which a type *T* subject would receive a *p* judgment by judge *j* is

$$R_{T_{j}}=1-F_{j}(l_{j}^{*}|T)$$ (14)

where $l_{j}^{*}$ represents judge *j*’s decision criterion defined in (3). ${\bar{R}}_{T}\equiv \sum_{j=1}^{J}w_{j}R_{T_{j}}$ is the unconditional probability of a type *T* subject receiving a *p* -judgment, given random judge assignment with assignment shares *w*_*j*_. The *p*-judgment rate, or propensity, of each judge is $\Psi_{j}$ as defined in (9). Thus, the average propensity of judges is $\bar{\Psi }\equiv \sum_{j=1}^{J}w_{j}\Psi_{j}$. I assume that there is at least some variation in judge propensities, i.e., that the *non-trivial instrument* requirement imposed in the literature holds, at least when judges hold no presumptions.

Using this notation, average monotonicity, which requires positive correlations between the likelihood of each type receiving a positive judgment and judge propensities, can be defined as follows (see [7]).

**Definition 3. (Average monotonicity).** Judge behavior satisfies average monotonicity if

$$z_{T}\equiv \sum_{j=1}^{J}w_{j}(\Psi_{j}-\bar{\Psi })(R_{Tj}-{\bar{R}}_{T})\geq 0\text{ for }T\in \{N,P\}$$ (15)

In intuitive terms, average monotonicity requires each subject’s likelihood of receiving a particular judgment to be increasing, on average, in his assignment towards judges who are more likely to make that judgment. Thus, average monotonicity is unlikely to hold when judges who make fewer type-1 errors are also likely to make fewer type-2 errors. This is because then, movements across judges that lead to higher correct *p*-judgments are also likely to lead to lower incorrect *p*-judgments. Thus, when the proportion of type *P* subjects is greater, it would be unsurprising for the likelihood of type-*N* subjects receiving a *p* judgment to be negatively correlated with judges’ *p*-judgment propensities.

When judges face symmetric signals and have no presumptions, an implication of proposition 1 is that a judge who commits fewer type-1 errors than another judge must also commit fewer type-2 errors than that same judge. This is because the two types of errors are then symmetric, and therefore they must co-move across judges. The next proposition shows that under these conditions, the intuition described above holds, and average monotonicity is violated.

**Proposition 2. (Average monotonicity violations).** Average monotonicity is violated when judges face symmetric signals and hold no presumptions.

*Proof*: When $l_{j}^{*}=1$ for all *j*, it follows per (13) that


$$\Psi_{j}=\mu (1-F_{j}(1|P))+(1-\mu )F_{j}(1|P)=\mu +(1-2\mu )F_{j}(1|P)$$

Thus,


$$\Psi_{j}-\bar{\Psi }=\mu +(1-2\mu )F_{j}(1|P)$$

$$-\underset{k=1}{\overset{J}{\sum }}w_{k}(\mu +(1-2\mu )F_{k}(1|P))$$

$$=(1-2\mu )\left(F_{j}(1|P)-\underset{k=1}{\overset{J}{\sum }}w_{k}F_{k}(1|P)\right)$$

Similarly,


$$R_{Tj}-{\bar{R}}_{T}=1-F_{j}(1|T)-\underset{k=1}{\overset{J}{\sum }}w_{k}(1-F_{k}(1|T))$$

$$=-F_{j}(1|T)+\underset{k=1}{\overset{J}{\sum }}w_{k}F_{k}(1|T)$$

Therefore,


$$z_{T}=(1-2\mu )\underset{j=1}{\overset{J}{\sum }}w_{j}\left(F_{j}(1|P)-\underset{k=1}{\overset{J}{\sum }}w_{k}F_{k}(1|P)\right)\left(-F_{j}(1|T)+\underset{k=1}{\overset{J}{\sum }}w_{k}F_{k}(1|T)\right)$$

for T∈{N,P}, i.e.,


$$z_{P}=-(1-2\mu )\underset{j=1}{\overset{J}{\sum }}w_{j}{\left(F_{j}(1|P)-\underset{k=1}{\overset{J}{\sum }}w_{k}F_{k}(1|P)\right)}^{2}=-z_{N}$$

The non-triviality of the instrument implies that $F_{j}(1|P)\text{⧸}=\underset{k=1}{\overset{J}{\sum }}w_{k}F_{k}(1|P)$ for some judges and that (1−2μ)⧸=0, since, otherwise, $\Psi_{j}=\bar{\Psi }$ for all *j*. Therefore, either *z*_*P*_<0, or *z*_*N*_<0. □

Next, I construct a simple example to illustrate the intuition behind proposition 2.

**Example 1. (Liability determination by judges)**

Consider a population of 3000 defendants whose cases are randomly assigned to judges J={1,2,3}. Each judge is tasked with determining whether the defendant is liable in each case assigned to them. For simplicity, suppose that liability hinges on whether the defendant was negligent (in which case she ought to be found liable) or diligent (in which case she ought to not be found liable). The proportion of negligent defendants is 60%. Because judges receive symmetric signals, as noted via proposition 1, they make symmetric errors; and $R_{N_{1}}=0.05;$ $R_{N_{2}}=0.3$; and $R_{N_{3}}=0.45$. Thus, judges are ordered according to their type-1 errors: Judge 1 makes the least amount of errors while judge 3 makes errors very frequently. Suppose further that misclassifications made by each judge in fact equal the expected number of classifications given this structure. Each judge’s propensity of finding liability is then given by:


$$\Psi_{j}=\frac{\text{\# Correct Liability + \# False liability}}{\text{ 1000}}$$

$$=\frac{\left(0.6\times R_{P_{j}}\times 1000)+(0.4\times R_{N_{j}}\times 1000\right)}{1000}$$

The correct and false positive rates as well as the liability determination propensity of each judge is summarized in Table 4.

**Table 4:** Judge decisions in example 1.

*j*	$R_{P_{j}}$	$R_{N_{j}}$	# Cor. L.	# Fal. L.	$\Psi_{j}$
1	0.95	0.05	570	20	0.59
2	0.7	0.3	420	120	0.54
3	0.55	0.45	330	180	0.51

That the decisions of these judges violate average monotonicity can be verified through a simple inspection of Table 4: While the likelihood with which negligent individuals are found liable co-move in the same direction as the judges’ propensities; the opposite is true for diligent individuals. This can be seen by noting that the numbers in the $R_{N_{j}}$ column and the numbers in the $\Psi_{j}$ column are moving in opposite directions as one goes down along the rows.

A simple extension of this example can be used to describe how undetected average monotonicity violations can give rise to misleading claims in studies employing the judges design. In particular, suppose that the researcher is interested in how liability determinations affect the future diligence of individuals. Suppose that each type *N* individual (i.e., diligent in the present) is likely to be negligent in the future with probability 0.1, if not found liable in the present; but that this probability is increased to 0.2 if they are found liable despite their diligence in the present. Type *P* individuals (i.e., negligent in the present), on the other hand, are likely to be negligent in the future with probability 0.8 regardless of the judge’s decision today. Thus, a finding of liability increases future negligence for type *N* individuals and has no impact on type *P* individuals. Therefore, in reality, the impact of a finding of liability on future negligence is positive. However, the judges design would yield the opposite result. This is because the outcome difference across any two judges has the opposite sign as the propensity difference between the same two judges. To verify this, note that the proportion of defendants assigned to judge *j* who act negligently in the future is given by:

$$Y_{j}=(\mu \times 0.8)+(1-\mu )((R_{N_{j}}\times 0.2)+((1-R_{N_{j}})\times 0.1))$$ (16)

$$=0.52+0.04R_{N_{j}}$$

Thus, $Y_{1}<Y_{2}<Y_{3}$, but $\Psi_{1}>\Psi_{2}>\Psi_{3}$. In fact, it is easy to verify that the Wald ratio associated with any judge pair *i*,*j*, equals $\frac{Y_{i}-Y_{j}}{\Psi_{i}-\Psi_{j}}=-0.2$. Therefore, a judges design estimand, which corresponds to a weighted average of Wald ratios across different judge pairs, would be negative. It is important to note that this sign-reversal problem (as previously noted, e.g., in [11, 12], and [8]) can persist even when a very small proportion of cases lead to monotonicity violations. To see this, note that the sign reversal emerges in the example studied even when the proportion of type *N* individuals (i.e., 1−μ) is replaced with a very small number; and these are the individuals for which monotonicity violations are observed.

The result reported in proposition 2 easily extends to cases where judges have non-extreme or weak presumptions. This is formalized through the following corollary.

**Corollary 1. (Violations with weak presumptions).** Average monotonicity is violated when judges face symmetric signals and have weak presumptions, i.e., there exists $\bar{\rho }>0$, such that $\rho_{j}<\bar{\rho }$ for all *j* implies that average monotonicity is violated.

*Proof*: *z*_*P*_ and *z*_*N*_ are both continuous in $l_{j}^{*}$ for all *j* around $l_{j}^{*}=1$. Thus, there exists $\bar{\rho }>0$ such that $z_{P}z_{N}<0$ as long as $\rho_{j}<\bar{\rho }$ for all *j*. □

It is worth noting that the analysis here does not require a clear ordering of judges’ abilities in rendering judgments accurately as in the Blackwell [17] informativeness sense. This ordering would require that some judges are capable of producing a lower type-1 error given any targeted type-2 error compared to other judges. Instead, the judges considered here may or may not be Blackwell-comparable. This greatly increases the class of evidence generating processes that judges may be facing, and only imposes the requirement of symmetry. In fact, corollary 1 can be further extended to allow for slight asymmetries in evidentiary processes: average monotonicity is violated when judges have weak presumptions unless they face sufficiently asymmetric signals. Because this claim is fairly intuitive, I refrain from formalizing it, since this requires the introduction of a measure of asymmetry and tedious notation.

### 2.6 Judges with (strong) presumptions

Beyond the observations made via proposition 2 and corollary 1, it is difficult to identify additional general conditions under which average monotonicity would fail, without imposing additional restrictions, e.g., on the signals observed by judges or the distribution of judge presumptions. In this section, I identify an additional symmetry property regarding the distribution of presumptions across judges, which can be imposed to identify additional intuitive results in cases where judges may have strong presumptions. In particular, when judges may have presumptions of any strength, but these presumptions are symmetrically distributed across judges with otherwise similar characteristics, one can track the impact of variations in presumptions versus variations in judge performance in an intuitive manner. This distribution restriction can be formalized through the following definition.

**Definition 4. (Symmetric presumption distribution).** Judge presumptions are symmetrically distributed if any judge *j* with $l_{j}^{*}\text{⧸}=1$, has a symmetric counterpart: a judge $j^{\prime }$ with otherwise identical characteristics (i.e., $w_{j}=w_{j^{\prime }}$, and $f_{j}(.|.)=f_{j^{\prime }}(.|.)$) who holds a presumption of equal strength in the opposite direction, i.e., $l_{j^{\prime }}^{*}=1/l_{j}^{*}$.

The symmetric presumption distribution allows a systematic analysis of the impact of variations in presumptions versus variations in the performance of judges. In particular, with a symmetric presumption distribution, one can pair each judge who holds a presumption with their symmetric counterpart. Then, for each judge *j*, one can define a group to which the judge belongs as

$$g_{j}\equiv \begin{array}{ccc} \frac{R_{N_{j}}+R_{N_{j^{\prime }}}}{2} & \text{if} & l_{j}^{*}\text{⧸}=1\\ R_{N_{j}} & \text{if} & l_{j}^{*}=1 \end{array}$$ (17)

This grouping exercise brings together all judges without presumptions together with all pairs of judges who collectively have the same judgement characteristics. Thus, it follows that *E*(*R*_*N*_|*g*) = *g*. Moreover, due to the symmetry of signals and presumption distributions it follows that *E*(1−*R*_*P*_|*g*) = *g* (see the proof of proposition 3 for a verification of this claim). Thus, both expected error rates within each group equals the group’s label, or index, *g*. This allows a ranking of groups according to their judgment performances, with smaller *g* indicating more accurate decision making. However, the same type of ranking cannot be made across judges within the same group, since within each group judges with larger presumptions will have smaller *R*_*N*_ and larger $1\;-\;R_{P}$, and vice versa. This categorization and its properties are what allow one to compare variations in decisions based on performance differences across groups of judges against variations in decisions based on differences in presumptions within groups. These comparisons are related to average monotonicity violations, as follows.

**Proposition 3. (Violations with symmetric presumption distributions).** Suppose judges face symmetric signals and their presumptions are symmetrically distributed. Then, average monotonicity is violated if, and only if,

$$E(var(R_{N}|g))<var(g)|1-2\mu |$$ (18)

*Proof*: See Appendix A in Supporting information. □

Proposition 3 extends the analysis to circumstances where judge presumptions may be of various strength. In intuitive terms, it shows that average monotonicity is more likely to fail when there are relatively large variations in judge performance; and more likely to hold when there are relatively large variations in judge presumptions. Stated differently, it shows that whether average monotonicity holds depends on whether differences in judge presumptions within groups (captured by the left hand side) or differences in judge performance across groups (captured by the right hand side) account for more variation in judge decisions. If the latter accounts for more variation, average monotonicity fails, and vice versa.

The rationale behind this result is that as one moves subjects towards judges with smaller *g*, their probability of *p* judgments is reduced if they are type *N* but increased if they are type *P* subjects. The judge group’s propensity thus moves in the opposite of one of these directions. This dynamic pushes in the direction of average monotonicity violations. In fact, when all judges lack presumptions, all judge groups are singletons. Therefore, the dynamic described is the only one present, which leads to monotonicity violations as noted in Sect 2.5. On the other hand, as one alters the presumption of a judge while keeping all else equal, the judge’s propensity (Ψ) as well the probabilities of a *p* judgment for both types (*R*_*P*_ and *R*_*N*_) move in the same direction. This is because a judge who is more lenient (resp. stricter) makes fewer (resp. more) p decisions, regardless of subject type. Since two judges within the same group only differ from each other with respect to their presumptions, these variations make it more likely for average monotonicity to hold; the co-movement of propensities and *p*-judgment probabilities in the same direction is exactly what is required by average monotonicity.

## 3 Bounds and presumptions

The analyses in Sect 2 identify conditions under which average monotonicity is violated. Judge presumptions play a key role in these analyses, which reveal that when judge presumptions are weak, average monotonicity violations are more likely. Moreover, even when some judges possess strong presumptions, violations of average monotonicity may be observed when the variation across judge performance, in terms of the errors committed by them, are large compared to decision variations caused by differences in presumptions. This raises two related questions. First, can one conduct a bounding exercise regarding the type of variation in presumptions that must exist for average monotonicity to hold? And, second, can one identify judge presumptions to ascertain whether the true presumption distribution of judges possesses such variation? In Sects 3.1 and 3.2, I answer the former question; and in Sect 3.3, I comment on the latter question.

### 3.1 Bounds on error rates

A bounding exercise can be conducted to interpret what level of variation in decisions due to presumption differences is necessary, given the assumptions made here, to ensure that average monotonicity holds. This type of exercise becomes possible when one specifies the distribution of errors committed by judges (i.e., $R_{N_{j}}$) in a tractable way. For instance, using the notation and framework introduced in Sect 2.6, when both average errors committed by groups (i.e., *g*) and the errors committed within groups (i.e., *R*_*N*_|*g*) are distributed uniformly, one can identify the maximum variation across judge errors due to differences in presumptions that lead to average monotonicity violations. In particular, suppose *g* is distributed uniformly with support [0,0.5]. (Note that *g*_*j*_>0.5 is not possible as this would imply that $F_{j}(l|N)<F_{j}(l|P)$, which contradicts the properties of *F* previously described. The case of *g*_*j*_ = 0.5 would be obtained when judges in group *g*_*j*_ make decisions as poorly as a random draw.) Suppose further that *R*_*N*_|*g* is distributed uniformly with support [(1−k(g))g,(1+k(g))g]. Here, *k*(*g*) denotes the maximum presumption-based-deviation of judge error from his group’s mean measured in proportional terms (i.e., $k(g)=\frac{\left|R_{N_{j}}-g\right|}{g}$). This deviation is expressed as a function of *g* to allow deviations to depend on the group. It is also worth noting that judges are assumed to form a continuum (as opposed to a discrete set) to simplify derivations.

Under these assumptions, by using the simple properties of the uniform distribution, one can verify that the condition in (18) holds as long as

$$\bar{k}\equiv \sqrt{\frac{3}{4}|1-2\mu |}>k(g)\text{ for all }g$$ (19)

For instance, $\bar{k}=50\%$ when μ=1/3. This implies that, even when the difference in errors caused by two judges’ differences in propensities are as large as their average error, average monotonicity is violated. Thus, $\bar{k}$ is an upper-bound on deviations such that $k(g)<\bar{k}$ for all *g* implies that average monotonicity is violated. Quite importantly, $\bar{k}$ specifies an upperbound on deviations in terms of decision errors as opposed to judge presumptions. A similar bounding exercise for presumptions is conducted, next.

### 3.2 Bounds on presumptions

One aspect of proposition 3 and the exercises in Sect 3.1 is that they focus on variation in judges’ error rates caused by presumptions as opposed variation in judges’ presumptions. The manner in which these errors are translated into judge presumptions is dictated by the properties of the signal generation processes used by judges. In general, the symmetry of the signal obtained by a judge with $R_{N_{j}}<1\;-\;R_{P_{j}}$ implies that he may not hold a presumption of *P*, but otherwise the presumptions of this judge may lie anywhere between 1 and $\frac{R_{P_{j}}}{R_{N_{j}}}$, i.e., $l_{j}^{*}\in [1,\frac{R_{P_{j}}}{R_{N_{j}}}]$. Stronger presumptions can be ruled out whenever the judge uses information efficiently (as in the Neyman-Pearson lemma sense), since then always choosing an *n* classification would be superior for that judge to choosing a decision criterion that leads to the error rates $R_{N_{j}}\;$and $1-R_{P_{j}}$. That any weaker presumption, i.e., any presumption $l_{j}^{*}\in [1,\frac{R_{P_{j}}}{R_{N_{j}}}]$ can be held by the judge is illustrated in Appendix B in Supporting information, by considering a judge who receives a symmetric ternary signal (i.e., a signal with only three realizations, one of which is associated with a likelihood ratio of 1). Note, that the argument almost completely extends to cases where judges receive continuous signals, in which case any presumption $l_{j}^{*}\in (1,\frac{R_{P_{j}}}{R_{N_{j}}})$ could be supported.

This observation can be combined with the simple example provided in Sect 3.1 to conduct a bounding exercise on judge presumptions rather than decision errors. In particular, it follows from (17) that the upper-bound on $l_{j}^{*}$ for a judge with $R_{N_{j}}<1-R_{P_{j}}$ is given by

$${\bar{l}}_{j}\equiv \frac{R_{P_{j}}}{R_{N_{j}}}=\frac{1-2g_{j}+R_{N_{j}}}{R_{N_{j}}}=1+\frac{1-2g_{j}}{R_{N_{j}}}$$ (20)

where the first equality follows directly from (17). This, in turn, implies that the upper-bound on the strength of the presumption of judge *j*, as well as his symmetric counter-part $j^{\prime }$, is

$${\bar{\rho }}_{j}\equiv \ln({\bar{l}}_{j})$$ (21)

The cumulative distribution of $\bar{\rho }$, can be derived, as shown in Appendix C in Supporting information, by using the uniform distributions of *g* and *R*_*N*_|*g* previously assumed. For any deviation profile such that k(g)=k∈(0,1] for all *g*, this distribution is given by

$$Z(\bar{\rho },k)=1-\frac{2}{k(e^{\bar{\rho }}-1)}\ln(\frac{\left(e^{\bar{\rho }}+1\right)}{(1-k)(e^{\bar{\rho }}-1)+2})$$ (22)

with

$$Z_{k}(\bar{\rho },k)<0\text{ for all }\bar{\rho }\in (0,\infty )\text{; and}$$ (23)

$$Z_{0}(\bar{\rho })\equiv \underset{k\to 0}{\lim}Z(\bar{\rho },k)=\frac{e^{\bar{\rho }}-1}{e^{\bar{\rho }}+1}$$

**Fig 1:**
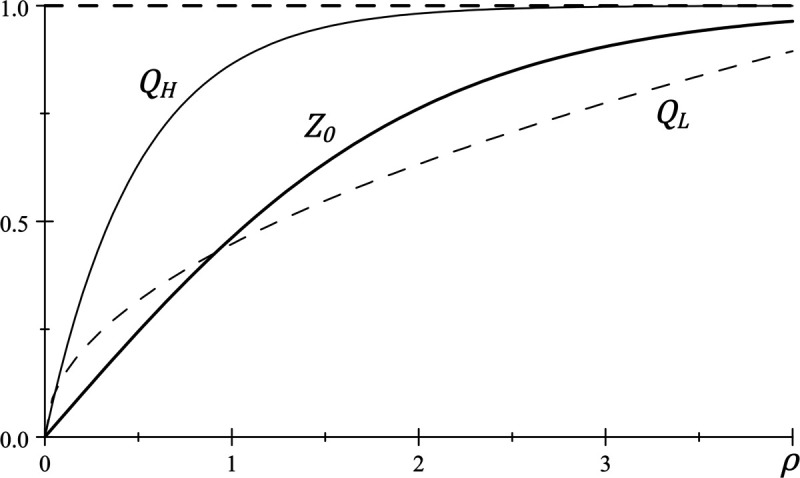
Presumption strength distributions.

Using these observations, and denoting the true presumption strength of judges as Q(ρ) one can make statements about when average monotonicity will hold, given the assumptions made thus far. In particular, if *Q* is first order stochastic dominated by *Z*_0_, then it follows that average monotonicity violations cannot be ruled out. This possibility is depicted in Fig 1, which plots $Z_{0}(\rho )$ (*thick*), and $Q_{H}(\rho )$ (*thin*) which is first order stochastic dominated by *Z*_0_. This follows, because a decision deviation profile with $k<\bar{k}(\mu )$ can then be supported through a presumption distribution that lies between the distributions $Z_{0}(\rho )$ and Pr(ρ=0)=1 (i.e., the dashed line depicting ρ=1 in Fig 1), which implies a violation of average monotonicity as noted via (19). This is because $Z(\rho ,\bar{k}(\mu ))$ and Pr(ρ=0)=1 are, respectively, the distributions of the upper-bound and lower-bound presumption strengths that judges may possess while generating errors consistent with $k(g)=\bar{k}(\mu )$ for all *g*. Because $Z(\rho ,\bar{k}(\mu ))<Z_{0}(\rho )$ for all ρ>0, it follows that *Q*_*H*_ lies between $Z(\rho ,\bar{k}(\mu ))$ and Pr(ρ=0)=1 as well. On the other hand, if *Q* is not first order stochastic dominated by *Z*_0_, it follows that for some range of priors, the true presumption profile is inconsistent with any presumption based judgment variation that is small enough for the inequality in (??) to hold. This possibility is depicted in Fig 1, through the distribution $Q_{L}(\rho )$ (*dashed*) which crosses $Z_{0}(\rho )$.

This exercise demonstrates how one could make statements about the conditions under which average monotonicity would hold, absent information regarding judges’ decision errors, and only by relying on the distribution of judge presumptions. Consistent with the analysis in Sects 2 and 3.1, it reveals that when judges have weak presumptions, average monotonicity violations cannot be ruled out; but also provides more precise bounds on presumption distributions that may lead to average monotonicity violations. Naturally, conducting this type of bounding exercise comes at the cost of loss of generality, as it requires the imposition of assumptions which were not imposed in Sect 2. Nevertheless, it provides an example of how researchers could conduct bounding exercises by focusing on the presumption distribution of judges; and the analysis can be repeated by replacing the specific assumptions imposed (e.g., regarding the intra- or inter-group distribution of judges, as well as the symmetry of judge presumptions) with others that researchers may find more suitable in the contexts they are studying.

### 3.3 Empirically identifying presumptions

The analyses presented highlight that average monotonicity may not hold when judges have weak presumptions, and that one can identify specific bounds in the form of presumption distributions. However, information regarding judge presumptions is not readily available. This raises the question of whether and how researchers can attempt to measure the presumptions of decision makers.

One possible way to obtain information about presumptions is by conducting surveys with judges whose decisions are being analyzed. In particular, judges can be asked to provide information regarding their preferences (e.g., through the question: How many type-1 errors is a type-2 error worth?) as well as their priors (e.g., through the question: What proportion of the subjects you encounter are type *P*?). The combined information can then be used to infer the decision maker’s presumptions. (Note that one could also pose a single question of the form: how strong must the evidence opposing your presumption be for you to render a decision in the opposite direction? However, formulating this question in a manner that is both easy to understand, and in a manner where the strength of the evidence referred to is measured in likelihood ratios may be difficult, if not impossible. Thus, one may instead seek to obtain information about judges’ preferences and priors, as described here, and combine them to make inferences regarding presumptions.)

Alternatively, one may design an experiment in which judges are asked to make decisions resembling the real-life decisions they make, and are informed (or asked to assume) that the proportion of type *P* cases they face in the experiment is similar to that they encounter in their real-life tasks. They could then be provided with, and educated about, a signal generating process, and subsequently asked about the threshold signal realization they would need to observe to return a *p* judgment. This information can then be used to measure judges’ presumptions.

## 4 Conclusion

A central condition in instrumental variables designs exploiting variation across judges’ propensities is average monotonicity. Detecting violations of this assumption is difficult, because the condition is related to the behavior of judges in counterfactuals, which are unobservable. Therefore, existing tests of average monotonicity cannot always be implemented, and they are capable of only detecting a subset of average monotonicity violations even when they are implementable. This raises the importance of having plausible theoretical rationales for assuming average monotonicity. The theoretical investigation provided here reveals two simple conditions under which average monotonicity is violated, namely symmetry and lack of strong presumptions. Thus, a prudent practice in studies employing the judges design may be to investigate, or at the very least discuss the plausibility of, judges having strong presumptions and/or facing asymmetric signals. Surveys or experimental studies geared towards identifying judges’ presumptions and using them for conducting bounding exercises, as outlined in Sect 3, therefore appear particularly promising. Studying the symmetry properties of signals reviewed by judges may prove to be a more challenging task, and scholars may thus have to resort to intuitive discussions of these properties. However, future work that builds on insights from the psychology literature focusing on similar issues (e.g., as in [18]) may have potential.

When one cannot confidently rule out violations of average monotonicity, it may be useful to provide explanations of how results obtained through judges designs would be altered in the presence of average monotonicity violations. A good example is the approach adopted in [13], wherein it is explained how results are affected by average monotonicity violations in the presence of specific hypothetical heterogeneities in treatment effects. Alternatively, one may resort to using empirical designs which require weaker or more plausible assumptions. For instance, monotonicity assumptions may be less suspect in IV approaches where the instrument has a more mechanical relationship to the potential treatment of subjects than in judges designs. To name an example, in recent work, Alsan et al. study the impact of an in-jail program called IGNITE on the behavior of incarcerated individuals; and “use court delays to instrument for the time a jailed person is exposed to IGNITE” ([19] p. 1369). There, monotonicity holds when exposure to the IGNITE program is (weakly) increasing in court delays, which appears to be a rather mechanical and plausible assumption. Another approach that can be taken is to consider tests which rely only on bounds on a measure of interest and therefore do not require monotonicity. [20], for instance, takes this approach, and proposes an absolute test of racial prejudice, and thereby circumvents the need to rely on the monotonicity assumption utilized in prior work to detect discrimination. Alternatively, researchers may investigate newer methods that attempt to identify judge pairs whose behavior, in fact, satisfy the monotonicity condition (see, e.g., [21]), and focus on the local average treatment effects associated with their decisions.

## Supporting information

S1 File Appendices for “Symmetry, Presumptions, and the Judges Design”. (PDF)
