# Correction: Symmetry, presumptions, and the judges design

**DOI:** 10.1371/journal.pone.0346832

**Published:** 2026-04-08

**Authors:** The PLOS One Staff

S1 File was uploaded incorrectly. Please see the correct S1 File. The publisher apologizes for the error.

## Supporting information

S1 File Appendices for “Symmetry, Presumptions, and the Judges Design”. (PDF)
