# Supplementary material for: Correction: Symmetry, presumptions, and the judges design
Source: PLoS One. 2026 Apr 8;21(4):e0346832. doi: 10.1371/journal.pone.0346832 (PMC13061234; doi:10.1371/journal.pone.0346832)
Supplement: S1 File — (PDF) [file pone.0346832.s001.pdf]

Appendices for "Symmetry, Presumptions, and the Judges Design"

Murat C. Mungan

January 16, 2026

Appendix A

Proof of Proposition 3

Using the law of total covariance one can re-write the terms in the definition of average monotonicity as follows:

$$z_T = E(cov(R_T, \Psi|g)) + cov(E(R_T|g), E(\Psi|g)) \qquad \text{(A.1.)}$$

This re-expression decomposes the covariance into two parts: intra-group covariance (i.e., the expected covariance within each group between R_T and Ψ), and inter-group covariance (the covariance between the expected R_T and Ψ of groups).

To simplify this expression, note, as a preliminary matter that the symmetry of the signal implies that

$$F_j(\frac{1}{l}|P) \ = 1 - F_j(l|N) \text{ for all } l > 0$$

since

$$F_j(\frac{1}{l}|T) = \Pr(L \leq \frac{1}{l}|T) = \Pr(\frac{1}{L} \geq l|T) = 1 - \tilde{F}_j(l|T)$$

and, per definition 2, it follows that $1 - \tilde{F}_j(l|P) = 1 - F_j(l|N)$. This property, in turn, implies that for j with $l_j^* \neq 1$ and his symmetric counterpart j'

$$R_{N_j} = 1 - R_{P_{j'}}$$

Thus, $E(R_N|g) = E(1 - R_P|g) = g$ which follows from the definition of g in section 2.6.

This observation is next used to re-write $E(\Psi|g) - \bar{\Psi}$ as

$$\begin{aligned} E(\Psi|g) - \bar{\Psi} &= \mu(E(R_P|g) - \bar{R}_P) + (1-\mu)(E(R_N|g) - \bar{R}_N) \\ &= \mu(\bar{R}_N - g) + (1-\mu)(g - \bar{R}_N) \\ &= (1-2\mu)(g - \bar{R}_N) \end{aligned}$$

Therefore, the inter-group covariance terms in (A.1.) become:

$$\begin{aligned} cov(E(R_N|g), E(\Psi|g)) &= \sum w_g(g - \bar{R}_N)(1-2\mu)(g - \bar{R}_N) \\ &= (1-2\mu)\sum w_g(g - \bar{R}_N)^2 \\ &= (1-2\mu)var(g) = -cov(E(R_P|g), E(\Psi|g)) \end{aligned}$$

where w_g is the share of subjects assigned to judges in group g. Similarly, the intra-group covariance can be written as:

$$\begin{aligned} E(cov(R_T, \Psi|g)) &= \sum w_g \sum_{j:g_j=g} w_j(R_{T_j} - E(R_T|g))(\Psi_j - E(\Psi|g)) \\ &= \sum w_g \sum_{j:g_j=g} w_j(R_{T_j} - E(R_T|g))^2 \\ &= E(var(R_T|g)) \end{aligned}$$

and $R_{P_j} - E(R_P|g) = R_{N_j} - E(R_N|g)$ due to the symmetry of judge presumptions, which also implies that

$$E(var(R_P|g)) = E(var(R_N|g))$$

Thus, $z_N \leq 0$ iff $E(var(R_N|g)) \leq (1-2\mu)var(g)$; and $z_P \leq 0$ iff $E(var(R_N|g)) \leq -(1-2\mu)var(g)$. Combining these two conditions reveals that average monotonicity is violated when $E(var(R_N|g)) < var(g)|1-2\mu|$, and it is not violated otherwise.■

Appendix B

Figure A.1. depicts the efficient decision frontier, frequently called the Receiver Operating Characteristics (ROC) curve, of a judge who receives a ternary signal in R_N,R_P space. In general, a symmetric ternary signal is of the form $X \in \{x_P, x_O, x_N\}$ with $\Pr(X = x_\tau|T = \tau) = q_1$, $\Pr(X = x_O|T = P, N) = q_2$, and $\Pr(X = x_\tau|T \neq \tau) = 1 - q_1 - q_2$ for $\tau \in \{P, N\}$. To construct an example, figure A.1. depicts the case where $q_1 = 0.6$ and $q_2 = 0.2$. The decision frontier is obtained through a straightforward application of the Neyman-Pearson lemma, and answers the following question: Suppose the judge wishes to target a specific type-1 error R_N, what decision criteria would he adopt to maximize R_P? This yields an efficient decision frontier of the form

$$\hat{R}_P(R_N) = \begin{cases} 3R_N & \text{if} \quad R_N \leq 0.2 \\ R_N + 0.4 & \text{if} \quad R_N \in (0.2, 0.4) \\ \frac{1}{3}(R_N - 0.4) + 0.8 & \text{if} \quad R_N \geq 0.4 \end{cases}$$

which is depicted in figure A.1.

Next, note that when judges' indifference curves are depicted in the same R_N, R_P space as the efficient decision frontier in figure A.1., their slopes represent their presumptions. This follows directly from the discussion of judge

utilities and presumptions in section 2.2. A number of indifference curves for a judge with presumptions $l_j^* = 2$ are depicted as dashed lines in figure A.1.. Judges' utilities are increasing towards the upper left part of the figure. Therefore, the judge chooses a point along his efficient decision frontier that places him on the highest indifference curve possible. Thus, for any presumption $l_j^* \in (1,3)$, the judge chooses the point A in figure A.1.. The judge choosing point A is also consistent with him having a presumption $l_j^* \in \{1,3\}$, because he would then be indifferent between choosing point A and others which yield the same utility.

Figure A.1.: Efficient Decision Frontier with a Ternary Signal

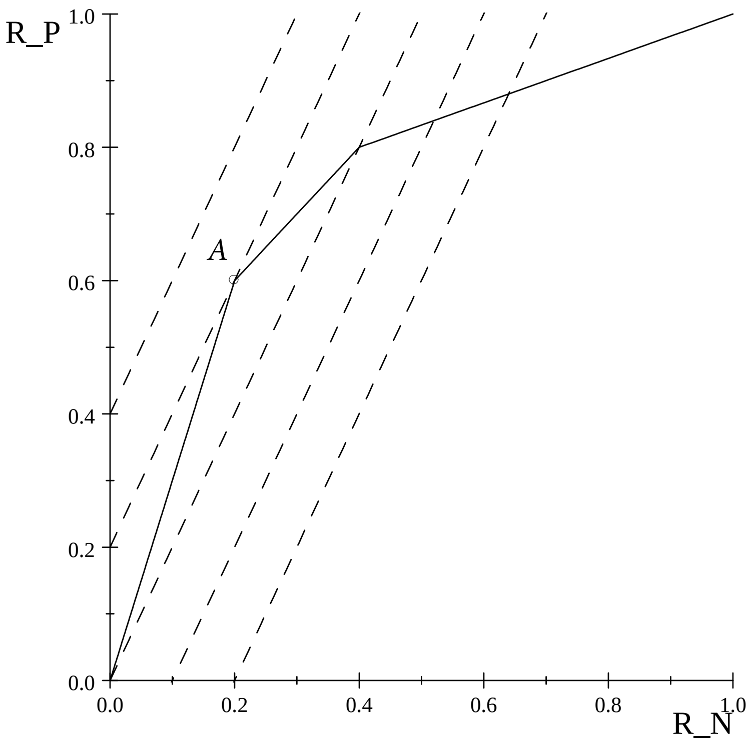

This example illustrates the more general point that a judge's decisions yielding the pair (R_{N_j}, R_{P_j}) is consistent with him having a presumption $l_j^* \in [1, \frac{R_{P_j}}{R_{N_j}}]$, since a judge receiving a ternary signal with $q_1 = R_{P_j}$ and $1 - q_1 - q_2 = R_{N_j}$ could make these decisions while possessing any presumption in the range $[1, \frac{R_{P_j}}{R_{N_j}}]$.

Appendix C

To derive the cumulative distribution of $\bar{\rho}$, first consider the distribution of $\bar{l}$ for $R_N < g$, i.e.,

$$\hat{Z}(l,k) \equiv \Pr(\bar{l} \leq l, R_N < g)$$

Using the definition of $\bar{l}$ in section 3.2. reveals that

$$\begin{aligned}\hat{Z}(l,k) &= \Pr(1 + \frac{1-2g}{R_N} < l, R_N < g) \\ &= \Pr(R_N \in [\frac{1-2g}{l-1}, g))\end{aligned}$$

Since g is uniformly distributed in $[0, 0.5]$ it follows that

$$\hat{Z}(l,k) = 2\int_0^{0.5} \Pr(R_N \in [\frac{1-2g}{l-1}, g)|g)dg$$

Noting that because $g < \frac{1-2g}{l-1}$ whenever $g < \frac{1}{l+1}$, it follows that

$$\Pr(R_N \in [\frac{1-2g}{l-1}, g)|g) = 0 \text{ for all } g < \frac{1}{l+1}$$

Moreover, it follows that for all

$$g > \hat{g}(l,k) \equiv \frac{1}{(1-k)(l-1)+2}$$

we have that $\frac{1-2g}{l-1} < (1-k)g$, and thus, $\Pr(R_N \in [\frac{1-2g}{l-1}, g)|g) = \frac{1}{2}$.

Therefore, $\hat{Z}(l,k)$ can be expressed as:

$$\begin{aligned}\hat{Z}(l,k) &= \int_{\frac{1}{l+1}}^{\hat{g}(l,k)} \frac{g - \frac{1-2g}{l-1}}{kg} dg + 0.5 - \hat{g}(l,k) \\ &= \frac{1}{k(l-1)}[(\hat{g}(l,k)(l+1) - 1) - \ln((l+1)\hat{g}(l,k))] + [0.5 - \hat{g}(k,l)]\end{aligned}$$

Noting that $\hat{g}(l+1) - 1 = k(l-1)\hat{g}$, it follows that

$$\begin{aligned}\hat{Z}(l,k) &= \frac{1}{k(l-1)}[k(l-1)\hat{g} - \ln((l+1)\hat{g})] + [0.5 - \hat{g}] \\ &= \frac{1}{2} - \frac{1}{k(l-1)} \ln(\frac{(l+1)}{(1-k)(l-1)+2})\end{aligned}$$

Finally, noting that $\rho(l) = \rho(\frac{1}{l})$, it follows that

$$Z(\bar{\rho},k) \equiv \Pr(\rho(\bar{l}) < \bar{\rho}) = 2\hat{Z}(e^{\bar{\rho}},k) = 1 - \frac{2}{k(e^{\bar{\rho}}-1)} \ln(\frac{(e^{\bar{\rho}}+1)}{(1-k)(e^{\bar{\rho}}-1)+2})$$
